# Lice Infestations in Sheep and Goats in Kombolcha District, East Hararghe Zone, Oromia Regional State, Ethiopia

**DOI:** 10.1155/2020/8889755

**Published:** 2020-12-24

**Authors:** Derara Dejene Disasa

**Affiliations:** Kombolcha Agricultural TVET College, Department of Animal Health, Oromia, Ethiopia

## Abstract

A cross-sectional study on ectoparasites of sheep (*n* = 134) and goats (*n* = 250) was conducted in Kombolcha district, east Hararge zone Oromia regional state, east Ethiopia, from November 2018 to April 2019 to determine the prevalence of major lice infestation of sheep and goats and the associated risk factors. Out of the examined animals (384), 56 (41.8%) sheep and 88 (22.9%) goats were infested with *Damalinia* and *Linognathus* species of lice, respectively. Ewes and does were 3.6 times more at risk for *Damalinia ovis* than rams and bucks, and young ones were 2.1 times more at risk than adults. Animals with poor body condition scores were 6.9 times more at risk for *Linognathus* species than those with good body condition small ruminants (*P* < 0.05). The observed overall prevalence is generally relatively high which may result in enormous economic losses through decreased production and productivity, damages to the skin, and deaths of the animal which requires an immediate professional and governmental attention.

## 1. Introduction

Ethiopia has a population of about 59.5 million cattle, 30.7 million sheep, and 30.2 million goats. However, the economic gains from these animals remain insignificant when they are compared to their huge number [1]. In Ethiopia, small ruminants constitute about 30% of the total livestock population of the country and are among important contributors to food production in Ethiopia, providing 35% of meat consumption and 14% of milk consumption [2]. At the national level, sheep and goats account for about 90% of the live animal/meat and 92% of skin and hide export trade value [3]. However, poor health and productivity of animals due to disease has become the major stumbling block to the potential of the livestock industry [4].

Lice are among the major diseases of small ruminants and cause serious economic loss to farmers through mortality, decreased production and reproduction, down grading, and rejection of skins which also affect the tanning industries [5]. Tanneries reported that 35% of sheep skins and 56% of goats' skins are rejected due to external parasites, and out of the reject groups of the processed skins, about 80 to 90% of the defects were believed to be due to external parasites. The estimated economic loss due to drop in the quality of sheep and goat skins is around USD 25.8 million per year [6].

All species of lice cause irritation of the skin, elicit scratching, rubbing, and licking leading to restlessness. These behaviors have a great effect on sheep production and skin quality [7]. Currently, there is a paucity of information regarding to lice infestation of small ruminants in Kombolcha district, east Hararge zone. Therefore, the main objectives of this study were to determine the prevalence of lice on small ruminants in and around Kombolcha district and identify lice and the associated risk factors involved in small ruminants in the study area.

## 2. Materials and Methods

### 2.1. Study Area

Kombolcha is one of the eighteen districts (woredas) in the east Hararghe zone which is located about 514 km from Addis Ababa and 14 km northwest of Harar town, the capital of the Harare people Regional state. The altitude of the woreda (district) ranges between 1600 and 2400 meters above the sea level. The district is bordered by the Dire Dawa City Administration to the North, Harari Regional State to the South, Jarso district to the East, and Haramaya district to the West. The district has a rugged topography with many valleys. The northern part of the district is mountainous with steep slopes. These natural features limit accessibility to some of the kebeles (administrative boundaries). Typically, the district lies in lowland and midland areas. Rainfall is mainly bimodal, but it can be erratic as well as the main rainy season is from February to mid-May and from July to the end of August. Annually, the district receives a mean rainfall of 600–900 mm [8]. According to CSA [9], the national census reported a total population for this woreda of 140,080, of whom 70,967 were men and 69,113 were women. Kombolcha district has 86,942 cattle, 79,804 sheep, 82,873 goats, 273 camels, 228 poultry, 23,905 donkeys and 2 Horses.

### 2.2. Study Animals

The study animals were sheep and goats of both sexes and different age groups (young and adult) in and around Kombolcha district.

### 2.3. Sample Collection

The survey of lice was conducted on small ruminants of varying ages and genders. Collection of lice was conducted after proper restraining of the animals. The adult lice were manually collected from the body surface by hand and brush or comb. Hair coat was parted and examined for lice on five regions of the body surface, namely, head, neck, thoracic, abdominal, and tail regions, both on the right and left sides of these areas. The collected parasites were preserved in properly labeled plastic containers containing 70% ethanol. The collection bottles were labeled with serial numbers, while other data were written on the specified register format prepared for this particular purpose (date, address, sex, age, and species). The samples were then transported to a veterinary laboratory for further identification. Identification of the collected lice was performed by means of stereo- and compound microscope by appreciation of its mouth part according to the procedure described by Wall and Shearer [10].

### 2.4. Study Design

The study was conducted using a cross-sectional study design to determine the prevalence of small ruminants' lice. The sample was collected from small ruminants and kept under extensive production system. The lice were randomly collected from sheep and goats of different sex, body condition score, and age group (young under one year of age and adult above one year of age for both sheep and goats by considering the rate of eruption of teeth) [11, 12].

Since no studies have been done on the lice of small ruminants in and around Kombolcha district in particular, 50% was taken as approximate expected prevalence. So, the sample size was calculated according to Thrusfield [13]: sample size calculation, ninety-five percent confidence levels, 5% precision, and 50% expected prevalence were used for the computation. Though the required sample size was computed to be 384, a total of 384 (134 sheep and 250 goats) of different species, age, and sex group were examined to increase the precision of investigation:

(1) $$\begin{array}{c} N=\frac{1.96{}^{2}\text{pex}\left(1-\text{pex}\right)}{D2}, \end{array}$$

where, *N* = required sample size, pex = expected prevalence, and *D* = precision.

### 2.5. Data Analysis

The collected data were first entered and managed into Microsoft Excel worksheet and analyzed by statistical software, namely, SPSS version 22. Prevalence was determined by the formula described by Thrusfield [13] as the rate of number of infested animals and total number of animals in population. Associations between explanatory variables (species of animals, age, and sex) and prevalence were done by chi-square test, and *P* < 0.05 was set to indicate signif icance.

## 3. Results

The overall prevalence of lice in present study was 37.5%. Among 384 examined small ruminants, 56/134 (41.8%) of sheep and 88/250 (35.2%) of goats were infested with *Damalinia* and *Linognathus* species of lice, respectively (Table 1).

High prevalence of *Linognathus* lice infestation in young about 2.1 times than in adults and in higher in poor body condition 6.9 times than in good body condition score small ruminants (*P* < 0.05) was recorded (Table 3).

High prevalence of overall lice infestation in female about 1.96 times than in male, 2.0 times in young than in adults, and higher in poor body condition 7.0 times than in good body condition score small ruminants (*P* < 0.05) was recorded (Table 4).

## 4. Discussion

The overall prevalence of lice infestation in small ruminants was 37.5%, and 41.8% in sheep and 22.9% in goat infestation of lice was recorded. The result is lower than 51.4% and 42.2 % in sheep and goats, respectively, in eastern Ethiopia [14]. But this result is higher than the prevalence recorded in Tigray 1.3% and 6.1% in sheep and goats, respectively [15] (25.8% and 14.9% in sheep and goats, respectively).

The present study showed that high overall prevalence of lice infestation in female, young, and poor body condition score of sheep and goats (*P* < 0.05) was recorded than male, adult, and good body condition score, and it was similar to the report of Amare et al. [16] that reported sheep poor in body condition were 1.9 times more at risk for *Damalinia ovis* than good body condition sheep and goats poor in body condition were 3.5 times more at risk for *Linognathus* species than good body condition goats (*P* < 0.05).

## 5. Conclusion

The present study showed that *Damalinia* and *Linognathus* species of lice are infesting sheep and goats, respectively, in the study area. It was shown that two species of lice were the major small ruminants' pests. The study revealed that lice of small ruminants were widely distributed and prevalent in both sexes, body condition score, and in all age groups of small ruminants in the study area. The observed overall prevalence is generally high which will result in high economic losses through decreased production and productivity, deaths of the animal, and damages of the skin demanding an immediate attention and professional intervention.

## Figures and Tables

**Table 1:** Prevalence of different genera/species of ectoparasites infestation in sheep and goats.

Genera of lice	Sheep (*n* = 134)	Goats (*n* = 250)	Total (*n* = 384)
	No positive (prevalence in %)	No positive (prevalence in %)	No positive (prevalence in %)
*Damalina* species	56 (41.8)	0 (0.0)	56 (14.6)
*Linognatus* species	0 (0.0)	88 (35.2)	88 (22.9)
Total	56 (41.8)	88 (22.9)	144 (37.5)

High prevalence of *Damalinia* lice infestation in female about 3.6 times than in male small ruminants (*P* < 0.05) was recorded in Table 2.

**Table 2:** Logistic regression of associated risk factors with prevalence of *Damalinia* species of lice in small ruminants.

Variables in the equation		B	S.E.	Wald	Df	Sig.	Exp (B)	95% CI for Exp (B)	
								Lower	Upper
Step 1^a^	Sex (1)	1.279	0.537	5.662	1	0.017	3.592	1.253	10.299
	Age (1)	0.158	0.328	0.233	1	0.630	1.171	0.616	2.226
	BCS			3.388	2	0.184			
	BCS (1)	0.521	0.345	2.273	1	0.132	1.683	0.855	3.313
	BCS (2)	0.187	0.374	0.251	1	0.616	0.829	0.399	1.725
	Constant	−2.973	0.541	30.171	1	0.000	0.051		

^a^Variables included: species of animals, sex, age, and BCS.

**Table 3:** Logistic regression of associated risk factors with prevalence of *Linognathus* species of lice in small ruminants.

Variables in the equation		*B*	S.E.	Wald	Df	Sig.	Exp (B)	95% CI for Exp (B)	
								Lower	Upper
Step 1^a^	Sex (1)	0.141	0.338	0.173	1	0.677	1.151	0.593	2.234
	Age (1)	0.745	0.282	7.008	1	0.008	2.107	1.213	3.658
	BCS			36.468	2	0.000			
	BCS (1)	1.928	0.319	36.421	1	0.000	6.873	3.675	12.854
	BCS (2)	0.835	0.318	6.898	1	0.009	2.304	1.236	4.295
	Constant	−2.319	0.363	40.722	1	0.000	0.098		

^a^Reference: sex (male), age (adult), BCS (good).

**Table 4:** Logistic regression of associated risk factors with prevalence of overall Lice infestation in small ruminants.

Variables in the equation		*B*	S.E.	Wald	Df	Sig.	Exp (B)	95% CI for Exp (B)	
								Lower	Upper
Step 1^a^	Species of animals	−0.353	0.241	2.150	1	0.143	0.703	0.438	1.126
	Sex (1)	0.672	0.309	4.718	1	0.030	1.957	1.068	3.588
	Age (1)	0.697	0.258	7.316	1	0.007	2.007	1.211	3.325
	BCS			41.022	2	0.000			
	BCS (1)	1.948	0.304	40.928	1	0.000	7.013	3.862	12.738
	BCS (2)	0.491	0.267	3.393	1	0.065	1.635	0.969	2.758
	Constant	−1.574	0.356	19.594	1	0.000	0.207		

^a^Reference: species of animals (goats), sex (male), age (adult), BCS (good).

## Data Availability

The data used to support the findings of this study are available from the corresponding author upon request.

## References

[1] Central Statistical Agency (CSA) (2017). Agricultural sample survey. report on livestock and livestock characteristics. *Volume II Statistical Bullrtin*.

[2] Asfaw B. (1998). *The Tanning Industry. Proceedings of Control of Sheep and Goat Skin Disease to Improve Quality of Hides and Skin*.

[3] Gizaw G. (2008). Sheep resources of Ethiopia: genetic diversity and breeding strategy.

[4] Mekonen B., Hussen I., Bedane B. (2001). The distribution of Ixodid tick in central Ethiopia. *Onderstepoort Journal of Veterinary Research*.

[5] Ashenafi H., Tolossa Y. H., Yebegaeshet M. (2013). Impact of sheep and goats ectoparasites on the tanning industry in Tigray Region, Ethiopia. *Ethiopian Veterinary Journal*.

[6] Yacob H. T. (2014). Ectoparasitism: threat to Ethiopian small ruminant population and tanning industry. *Journal of Veterinary Medicine and Animal Health*.

[7] Bayou K. (1998). *Overview of Sheep and Goat Skin Diseases, Treatment Trial for Improved Quality of Hides and Skins (Phase II)*.

[8] Arayaselassie A. (2018). The study of homegarden agrobiodiversity, practices of homegardening and its role for in-situ conservation of plant biodiversity in eastern Hararghe, Kombolcha town Oromia regional state Ethiopia, school of animal and range sciences, college of agriculture and environmental science, Haramaya university, Dire Dawa, Ethiopia. *Open Journal of Forestry*.

[9] CSA(Central Statistical Agency) (2007). Population census of Ethiopia. *Population and Housing Census Report-Country-2007*.

[10] Wall R., Shearer D. (1997). *Veterinary Entomonology*.

[11] Gatenby M. R., Coste R., Smith J. A. (1991). Sheep. *The Tropical Agriculturalist*.

[12] Steele M. (1996). *The Tropical Agriculturalist*.

[13] Thrustfield M. (2018). *Veterinary Epidemiolog,y*.

[14] Tamerat N., Zeryehun T. (2015). Prevalence and identification of ectoparasites fauna in small ruminants in selected areas of eastern Ethiopia. *African Journal of Basic and Applied Sciences*.

[15] Rahmeto A., Tatek M., Megersa B., Sheferaw D. (2011). Prevalence of small ruminants ectoparasites and associated risk factors in selected districts of Tigray region, Ethiopia. *Global Veterinarian*.

[16] Amare Sisay, Asfaw Y., Hailu Y. (2013). Ectoparasites of sheep and goats in north-west amhara regional state, Ethiopia. *Ethiopian Veterinary Journal*.

